# Interleukin-33 regulates intestinal inflammation by modulating macrophages in inflammatory bowel disease

**DOI:** 10.1038/s41598-017-00840-2

**Published:** 2017-04-12

**Authors:** Dong Hyuk Seo, Xiumei Che, Min Seob Kwak, Soochan Kim, Jae Hyeon Kim, Hyun Woo Ma, Da Hye Kim, Tae Il Kim, Won Ho Kim, Seung Won Kim, Jae Hee Cheon

**Affiliations:** 1 Department of Internal Medicine and Institute of Gastroenterology, Yonsei University College of Medicine, Seoul, Korea; 2 Brain Korea 21 PLUS Project for Medical Science, Yonsei University College of Medicine, Seoul, Korea; 3 Severance Biomedical Science Institute, Yonsei University College of Medicine, Seoul, Korea; 4 Department of Internal Medicine, Graduate School, Yonsei University College of Medicine, Seoul, Korea; 5 Department of Internal Medicine, Kyung Hee University Hospital at Gang Dong, Kyung Hee University School of Medicine, Seoul, Korea

## Abstract

Interleukin 33 (IL-33) that signals through the ST2 receptor has emerged as a critical modulator in several inflammatory disorders, including inflammatory bowel disease (IBD). However, the precise mechanisms by which IL-33 modulates IBD are controversial. The aim of this study was thus to clarify the role of IL-33 in IBD. The plasma levels of IL-33 were significantly decreased, but soluble ST2 levels were increased in patients with IBD compared to healthy individuals. Moreover, IL-33 restored goblet cell numbers and induced macrophage switching from the M1 to the M2 phenotype. These effects were sufficient to ameliorate colitis in dextran sodium sulfate, trinitrobenzene sulfonic acid, and peritoneal cavity cell transfer models. IL-33 facilitated goblet cell restoration via modulating macrophages toward the M2 phenotype. In addition, wound healing was significantly faster in IL-33-treated human monocyte-derived macrophages than in control cells, which could be attributed to increased polarisation into M2 macrophages. We found that patients with IBD show decreased serum levels of IL-33 compared with healthy individuals and that IL-33 can attenuate colitis and aid tissue repair in mice. The mechanism by which IL-33 exerts these effects appears to involve the stimulation of differentiation of goblet cells and M2 macrophages.

## Introduction

Inflammatory bowel diseases (IBDs) are chronic relapsing disorders of the intestine that are caused by complex interactions of environmental and genetic factors, along with subsequent changes in immune dysregulation. Recently, interleukin 33 (IL-33), a novel member of the IL-1 family and an alarmin cytokine that acts in response to various kinds of tissue damage^1^, has emerged as a critical modulator in several autoimmune and inflammatory disorders, including IBD. However, the role of IL-33 in IBD has not yet been clearly defined. Controversial data have been obtained regarding whether serum IL-33 levels correlate with IBD activity^2–4^. It is also a matter of controversy whether the IL-33 concentration is elevated in the inflamed intestinal lesions of patients with IBD^4–6^. Although there are several studies that have been performed in animal models to investigate the proinflammatory functions of IL-33 in colitis^7–9^, these studies have yielded conflicting results^9–14^. The role of IL-33 in the intestine seems to depend on the stage of inflammation, and can be either detrimental or protective^9, 13, 15^. Nevertheless, emerging evidence indicates that IL-33 plays a role in epithelial restoration, repair, and mucosal healing in ulcerative colitis (UC) and Crohn’s disease (CD)^11^.

Once the epithelial layer is damaged, it responds by restoring the continuity of the layer and the integrated structure via a complex regeneration process^16^. Goblet cells and macrophages play key roles in this tissue remodelling^17, 18^. Goblet cells, otherwise known as mucin secretory intestinal epithelial cells (IECs)^19^, form mucous layers and perform a critical barrier function. Classically activated macrophages (M1 macrophages) can be differentiated into alternatively activated macrophages (M2 macrophages), the latter of which are considered to produce only low levels of proinflammatory cytokines and to function more in the resolution of inflammation and tissue repair^20^. IL-33 has been reported to induce goblet cell hyperplasia both *in vitro* and *in vivo* through T helper 2 (Th2) cell expansions and through regulatory T cell (Treg) induction^8, 21^. IL-33 has also been shown to skew macrophages from the M1 to the M2 phenotype during airway inflammation^22^. However, these effects of IL-33 on goblet cells and macrophages have not yet been conclusively demonstrated in IBD.

Recently, it was reported that IL-33 colocalised with F4/80^+^ myeloid-derived cells in the inflamed intestinal lamina propria of mice in an oxazolone colitis model^10^. Given this finding, we speculated that IL-33 can directly modulate intestinal immune responses by driving the M1-to-M2 macrophage switch and macrophage-goblet cell cross-talk. Therefore, the aim of our study was to investigate the associations between IL-33 and IBD by exploring whether IL-33 contributes to the resolution of colitis and tissue repair by functional phenotype modulation of goblet cells and macrophages. To this end, we assessed the effect of IL-33 on colitis using well-established acute murine models of colitis. We also analysed clinical samples from patients with IBD.

## Results

### IL-33 expression is downregulated, but soluble ST2 expression is upregulated in serum of patients with IBD

**Figure 1:**
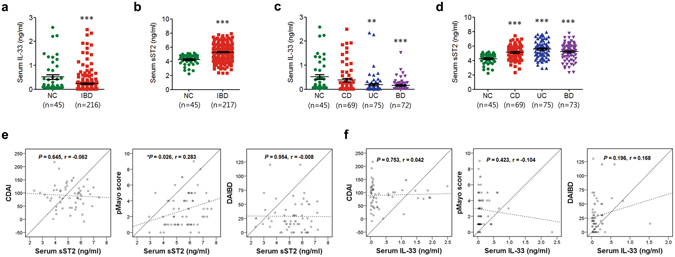
Levels of circulating IL-33 and soluble ST2 (sST2). Serum IL-33 and sST2 concentrations were determined in duplicate by enzyme-linked immunosorbent assay (ELISA). Two different anti-IL-33 antibodies were used, each of which recognized a different IL-33 epitope. The levels of IL-33 (**a**,**c**) and sST2 (**b**,**d**) were assessed by ELISA in serum samples from normal controls (NC) and patients with Crohn’s disease (CD), ulcerative colitis (UC), and Behçet’s disease (BD). The error bars represent the SEMs. (**e**,**f**) Correlations between serum IL-33 (**e**) and sST2 (**f**) levels and clinical disease activity of inflammatory bowel disease (IBD). **P < 0.01 vs. NC, ***P < 0.005 vs. NC as assessed by ANOVA. NC, normal control; CDAI, Crohn’s disease activity index; DAIBD, disease activity index for intestinal Behçet’s disease.

ST2, an IL-33 receptor, is mainly present as two forms: ST2L, which is membrane-bound, and sST2, which is soluble. sST2 has been shown to compete for IL-33 binding and to inhibit receptor signalling as a decoy receptor for IL-33^4^. Thus, we firstly examined circulating IL-33 and sST2 levels in serum samples from normal control subjects and patients with various IBDs, including CD, UC, and Behçet’s disease (BD), to determine whether IL-33 levels were correlated with IBD. For protein quantitation, we used an enzyme-linked immunosorbent assay (ELISA). The demographic and clinical characteristics of the patients with IBD are shown in Table S1. As shown in Fig. 1a−d, we found that the serum IL-33 levels (mean) in patients with IBD were lower than those in normal controls (*P* < 0.012: normal controls, 0.513 ± 0.665 ng/ml; IBD, 0.242 ± 0.436 ng/ml; CD, 0.381 ± 0.582 ng/ml; UC, 0.185 ± 0.394 ng/ml; intestinal BD, 0.160 ± 0.229 ng/ml), whereas the sST2 levels in patients with IBD were higher than those in normal controls (*P* < 0.001: normal controls, 4.246 ± 0.737 ng/ml; IBD, 5.344 ± 1.162 ng/ml; CD, 5.110 ± 1.054 ng/ml; UC, 5.581 ± 1.169 ng/ml; BD, 5.214 ± 1.174 ng/ml). These findings were consistent with those of several previous studies^2, 4^. In particular, patients with UC and intestinal BD showed significantly different serum IL-33 levels compared to healthy controls (*P* < 0.010 and *P* < 0.005, respectively). Moreover, the serum IL33/sST2 ratio was markedly reduced in patients with UC and patients with intestinal BD (Fig. S1a). Interestingly, these findings are the opposite of what was observed with serum IL-33 levels in patients with IBD. To evaluate whether IL-33 expression is up- or downregulated in the inflamed colon, we examined IL-33 gene expression levels in human and murine tissue samples from inflamed colons. Quantitative reverse transcription polymerase chain reaction (qRT-PCR) analysis revealed that IL-33 expression was upregulated in colonic mucosal samples from patients with IBD (Fig. S2a) and in colon tissue samples from dextran sodium sulfate (DSS)-treated mice (Fig. S2b) compared with the respective controls. We hypothesise that increased IL-33 levels during colitis might result from a compensatory mechanism against tissue damage by active inflammation itself. This increase might also result from secondary changes in response to other cytokines produced during inflammation^23^.

We next analysed disease severity by calculating various disease-appropriate activity scores. Linear correlation analysis revealed that the serum sST2 levels (r = 0.283, P = 0.026) were significantly associated with the pMayo score in patients with UC (Fig. 1e), whereas serum IL-33 levels were not (Fig. 1f). However, no significant correlations were observed between the serum IL-33/sST2 ratio and the partial Mayo scoring index (pMayo) score in patients with UC, the Crohn’s disease activity index (CDAI)^24^, in patients with CD, or the disease activity index in patients with intestinal Behçet’s disease (DAIBD)^25^ (Fig. S1b).

### IL-33 attenuates colitis via directly modulating goblet cell and macrophage functions

**Figure 2:**
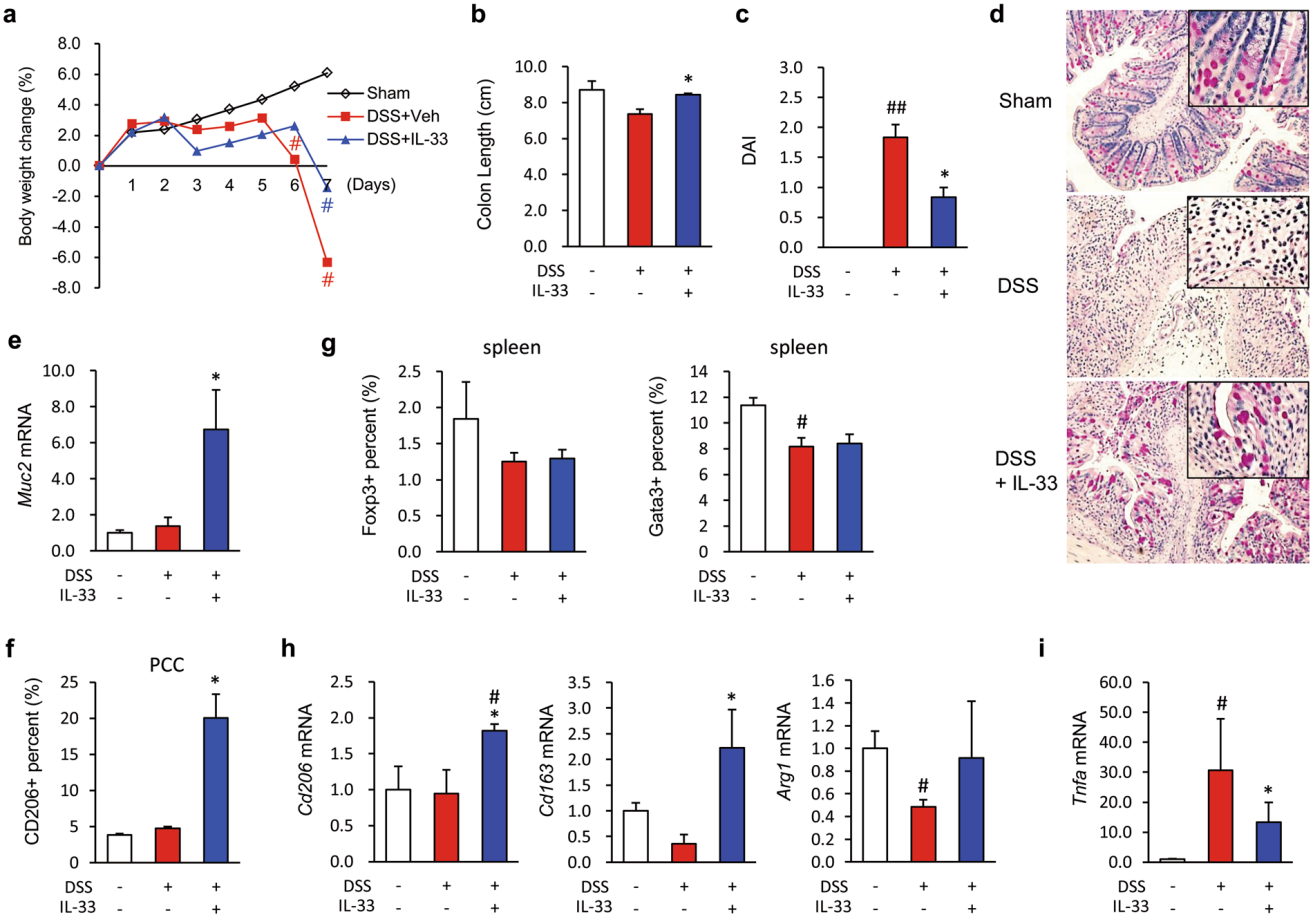
Effects of IL-33 on dextran sodium sulfate (DSS)-induced colitis in mice. (**a**) Body weight changes. The weight of each mouse was followed daily. (**b**) Effects of IL-33 on colon length. (**c**) Effects of IL-33 on the disease activity index (DAI). Disease activity at day 7 was scored according to the criteria outlined in the Materials and Methods. (**d**) Representative image of periodic acid-Schiff (PAS)-stained goblet cells in the colon. (**e**,**h**,**i**) mRNA expression in the mouse colon. mRNA expression of *Muc2* (**e**), M2 markers (*Cd206*, *Cd163*, and *Arg1*) (**h**), and *Tnfa* (**i**) were evaluated by quantitative RT-PCR. (**f**) Flow cytometry analysis of the M2 macrophage (CD206^+^) populations in peritoneal cavity cells (PCCs) from treated mice. (**g**) Flow cytometry analysis of the Treg (Foxp3^+^) and Th1 (Gata3^+^) populations in the spleens of treated mice. Data are presented as means ± SE (n = 5 mice/group). Recombinant IL-33 (mrIL-33) was i.p. injected daily (0.2 μg/mouse) for 5 days after treatment with 3% (w/v) DSS. ^#^ *P* < 0.05 vs. Sham control, *P < 0.05 vs. DSS as assessed by ANOVA.

Since IL-33 has been localised to enterocytes and the lamina propria of IBD patients and healthy controls^2, 4^, we hypothesised that IL-33 might directly modulate the differentiation of enterocytes and macrophages into goblet cells and M2 macrophages, respectively, but not through modulation of Treg and Th2. The pathogenesis of DSS-induced colitis, a model of UC-like disease, is considered to be driven by macrophages^26^. Therefore, to assess the effects of IL-33 on enterocyte and macrophage differentiation, mice were i.p. injected daily with mrIL-33 for 5 days after DSS treatment. In our study, mice in the IL-33-treated groups displayed significantly improved body weight recovery, longer colons, lower disease activity index (DAI) and histological score than mice in the vehicle-treated control groups (Figs 2a–c and S3). To explore the effects of IL-33 administration on goblet cells, we performed periodic acid-Schiff (PAS) staining. The colon sections from DSS-administered mice exhibited fewer goblet cells than colon sections from sham controls; moreover, more hypotrophic goblet cells containing less mucus were evident in the DSS colon sections. In contrast, the goblet cells showed near complete restoration in colon sections from the mrIL-33-treated DSS groups (Fig. 2d). Consistently, the mrIL-33-treated groups also showed increased mRNA expression of *mucin 2* (*Muc2*) in the colon; this gene encodes a major macromolecular constituent of intestinal mucus and is a known determinant of goblet cell morphology (Fig. 2e). We next examined whether IL-33 acts systemically on M2 polarisation of macrophages and T cell differentiation. To this end, we analysed the M2 population (CD206^+^) in the macrophages (F4/80^+^) from peritoneal cavity cells (PCCs) and the Treg (Foxp3^+^) and Th2 (Gata3^+^) populations in T cells (CD3^+^) from the spleens of sacrificed mice, respectively. PCCs from mrIL-33 + DSS-treated mice had a higher percentage of CD206^+^ macrophages compared with PCCs from vehicle + DSS-treated mice (Fig. 2f). As shown in Fig. 2g, however, the percentages of splenic Treg cells and Th2 cells were not significantly changed by mrIL-33 administration in the DSS-treated mice, contrary to the results of a previous study^8, 21^. These findings suggest that IL-33–mediated amelioration of colitis might not be associated with an enhanced Treg response in our mouse colitis models different from previous studies^27–29^, implying that other factors might contribute to the tissue protection observed after mrIL-33 treatment of colitis. We next hypothesised that IL-33 directly modulates goblet cells and macrophages to exert its protective effects against colitis. Consistent with this hypothesis, we observed upregulation of M2 markers such as *Cd206*, *Cd163*, and *Arg1*, but downregulation of M1 markers such as *tumour necrosis factor alpha* (*Tnfa*), in colon tissue samples from mrIL-33-treated mice (Fig. 2h,i).

**Figure 3:**
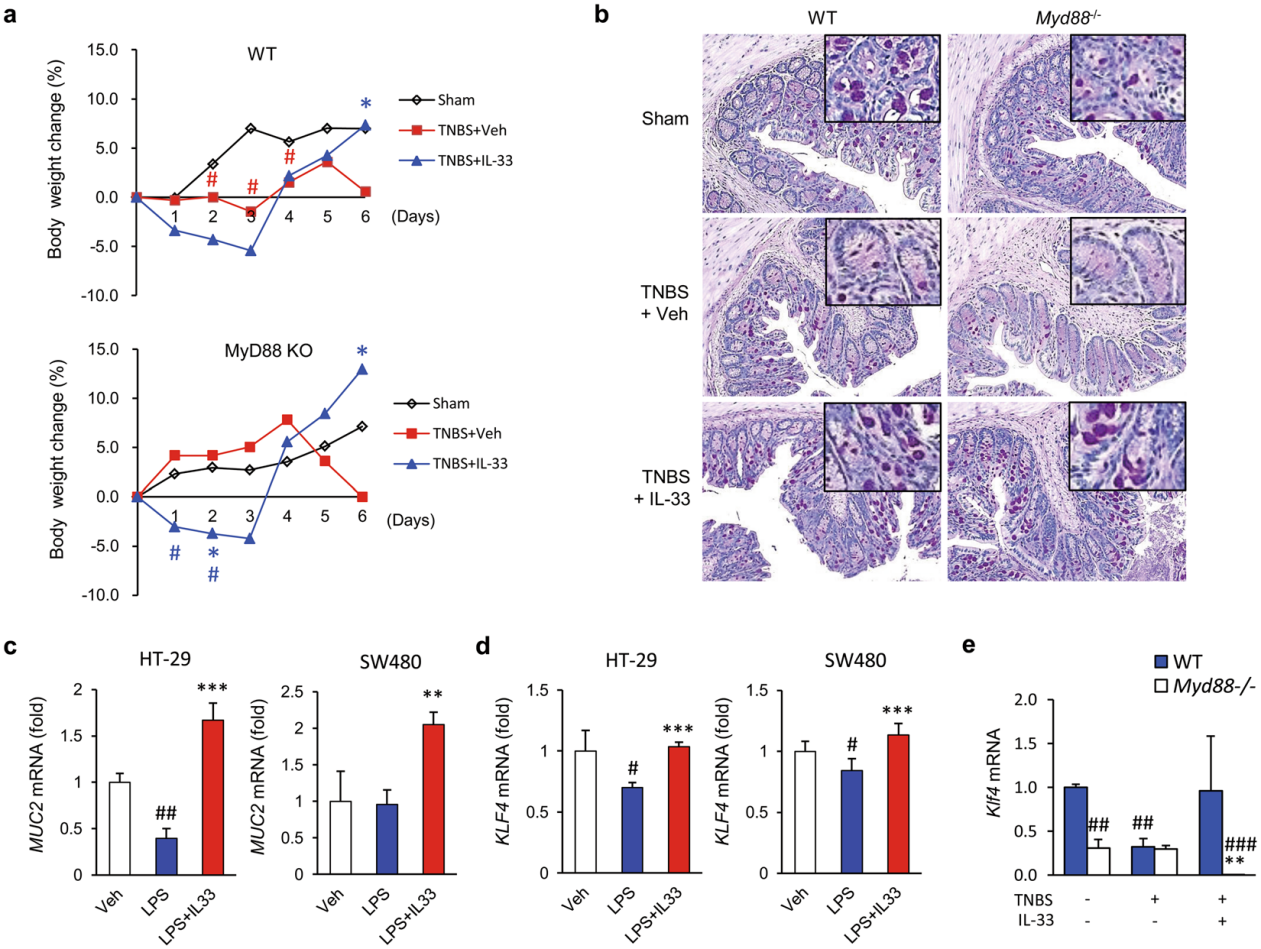
MyD88 independent effects of IL-33 on goblet cell restoration in inflammation. (**a**,**b**,**e**) Effects of IL-33 on colitis and goblet cells in the colon of wild-type (WT) and MyD88 deficient (*MyD88* ^−/−^) mice after low-dose 2,4,6-trinitrobenzenesulfonic acid (TNBS) administration (n = 5 mice/group). To assess the effects of IL-33 on enterocyte early in colitis, 100 µl TNBS solution (1.8% (w/v)) in 40% ethanol was injected into the colon of each mouse. (**a**) Changes in body weight. The weight of each mouse was followed daily. (**b**) Representative image of periodic acid-Schiff (PAS) staining of the colonic mucosa for goblet cells. Original magnification: x200. (**c**,**d**) Effects of IL-33 on *MUC2* (**c**) and *KLF4* (**d**) mRNA expression in human colonic epithelial cells. HT-29 cells were treated with vehicle (Veh) or lipopolysaccharide (LPS, 100 ng/ml), with or without human recombinant IL-33 (hrIL-33), for 4 hours. Total RNA was extracted and analysed by quantitative RT-PCR. Target mRNA expression levels were normalised to that of *β-actin*. Results are representative of two independent experiments. (**e**) Effects of IL-33 on *Klf4* mRNA expression in the mouse colon. Values are expressed as means; error bars represent ±SD. ^#^ *P* < 0.05 vs. Sham control, *P < 0.05 vs. TNBS or LPS, **P < 0.01 vs. TNBS or LPS, ***P < 0.005 vs. LPS as assessed by ANOVA. Veh, injected with vehicle; IL-33, once injected with mrIL-33 (1 μg/mouse) or hrIL-33 (20 ng/ml); TNBS, injected with TNBS.

Since the role of IL-33 in mouse colitis is controversial and IL-33 has been reported to inhibit Th1 and promote Th2 profiles^29^, we next evaluated the effect of IL-33 on another Th1-mediated CD-like colitis model using 2,4,6-trinitrobenzene sulfonic acid (TNBS). The mrIL-33-treated mice exhibited slightly longer life spans than the TNBS-treated mice, although this trend was not statistically significant (Fig. S4), suggesting that IL-33 exerts protective effects against Th1-mediated colitis. Although TNBS-induced colitis is Th1-mediated, the major source of IL-33 in the colonic tissues of TNBS-treated mice is infiltrated macrophages^11^. Moreover, IL-33 can signal via the myeloid differentiation primary response gene 88 (MyD88)-dependent pathway, which is important for the induction of mucosal inflammation at the onset of colitis^15^. To assess the effects of IL-33 on enterocyte differentiation early in colitis and the contribution of MyD88 to these effects, we i.p. injected wild-type or MyD88-deficient mice with mrIL-33 after low-dose TNBS administration. These mice were sacrificed before the development of severe colitis to investigate the effects of IL-33 at the onset of inflammation. In this setting, mrIL-33 administration restored body weight and reduced disease activity in both wild and MyD88-deficient mice (Figs 3a and S5a), although the body weight loss was induced by mrIL-33 administration in both groups in the early stage (until day 3) of colitis in agreement with the other study^9, 12, 13, 30^. These results suggest that IL-33 acts very early, before the development of severe colitis; moreover, they suggest that IL-33 acts independently of the MyD88 pathway. To explore changes in enterocytes after mrIL-33 administration in the setting of colitis development, we assessed goblet cells using PAS staining. Similar to what was observed in DSS-induced colitis, fewer goblet cells were observed in the colon sections from TNBS-administered WT and MyD88-deficient mice compared to sham controls; moreover, more of the goblet cells were hypotrophic. However, goblet cells in the mrIL-33-treated TNBS-treated group showed near complete recovery in both wild-type and MyD88-deficient mice (Fig. 3b). Consistent with these findings, colon tissue samples from TNBS-administered mice showed reduced mRNA expression of *Muc2*, whereas mrIL-33 administration markedly restored the mRNA expression of *Muc2* in the colon tissue samples from both groups of mice (Fig. S5b). These results suggest that goblet cell modulation by IL-33 is independent of the MyD88 pathway during colitis. To further confirm our *in vivo* findings, we examined the mRNA expression level of *MUC2* in colon IECs, HT-29 cells, and SW480 cells using qRT-PCR. Previous studies indicated that perturbed homeostasis between commensal bacteria and mucosal immunity, such as aberrant TLR modulation such as TLR4 by lipopolysaccharide (LPS), is an important stimulator of cytokines including TNF-α, may contribute to the development of gut inflammation and IBD^31–33^. LPS treatment reduces *MUC2* mRNA expression as previously described^34^. However, *MUC2* mRNA expression was consistently upregulated in hrIL-33-treated cells compared with the appropriate controls (Fig. 3c). Additionally, *Kruppel-like factor* (*KLF*) 4 mRNA, required for the terminal differentiation of goblet cells in the colon^35^, was upregulated in recombinant IL-33-treated cells (Fig. 3d) and in the colon of mice treated with TNBS compared with the controls (Fig. 3e). Taken together, our results indicate that goblet cell modulation by IL-33 might be directly influenced by IL-33 itself or by other non-T cells, such as macrophages.

### IL-33 directly switches macrophages from the M1 to the M2 phenotype; this switch is sufficient to ameliorate colitis and goblet cell differentiation

**Figure 4:**
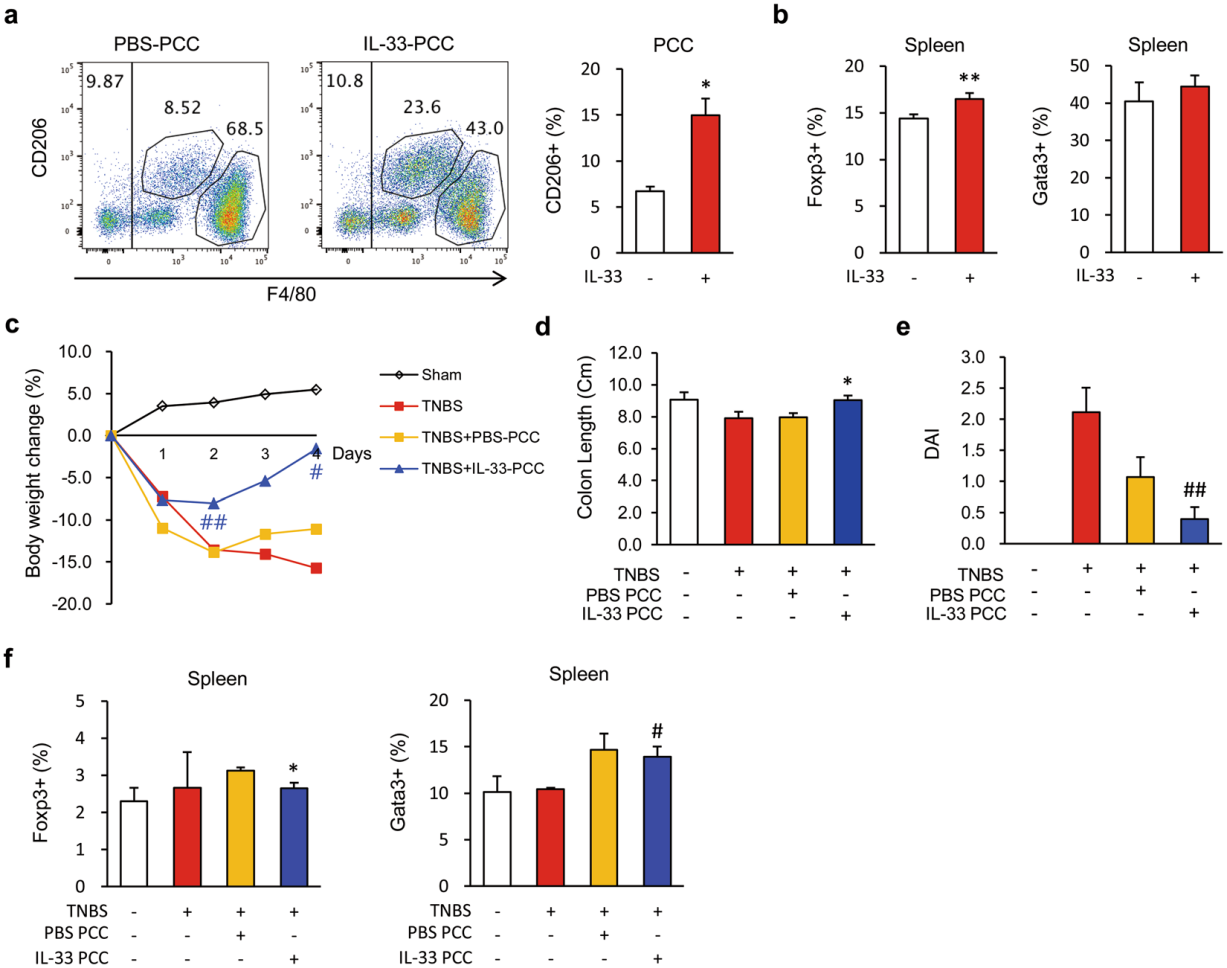
IL-33-treated peritoneal cavity cells ameliorate mouse colitis. Male C57BL/6 mice were inoculated i.p. with IL-33 (mrIL-33, 1 μg/mouse) or PBS for 3 consecutive days. Next, peritoneal cavity cells (PCCs) were extracted from the peritoneum of each mouse by washing the peritoneal cavity with cold PBS (**a**,**b**). F4/80 positive cells were sorted from the harvested PCCs and i.p. injected (1 × 10^6^ cells) into recipient mice. After 2 days, 2,4,6-trinitrobenzenesulfonic acid (TNBS) solution (2.2% (w/v)) was injected into the colon of each recipient mouse (**c**–**f**). (**a**,**b**) Representative flow cytometry plots of PCCs and spleen cells from IL-33-treated naïve mice. (**a**) Dot plot of the M2 (CD206^+^) macrophage (F4/80^+^) populations in the PCCs and percentages of the M2 macrophage populations in the PCCs. (**b**) Treg (Foxp3^+^) and Th1 (Gata3^+^) populations in the spleens of naïve mice. (**c**) Body weight changes of PCC-transplanted mice. (**d**) Colon lengths of PCC-transplanted mice. (**e**) Disease activity index (DAI) scores of PCC-transplanted mice. (**f**) Flow cytometry analysis of Treg (Foxp3^+^) and Th1 (Gata3^+^) populations isolated from the spleen of PCC-transplanted mice. Values are expressed as means (n = 5); error bars represent ±SD. ^#^ *P* < 0.05 vs. TNBS, ^##^ *P* < 0.01 vs. TNBS, *P < 0.05 vs. Veh or PBS PCC, **P < 0.01 vs. Veh or PBS PCC as assessed by ANOVA. Veh, injected with vehicle; IL-33, injected with mrIL-33; PCC, injected with F4/80^+^ PCCs; TNBS, injected with TNBS.

**Figure 5:**
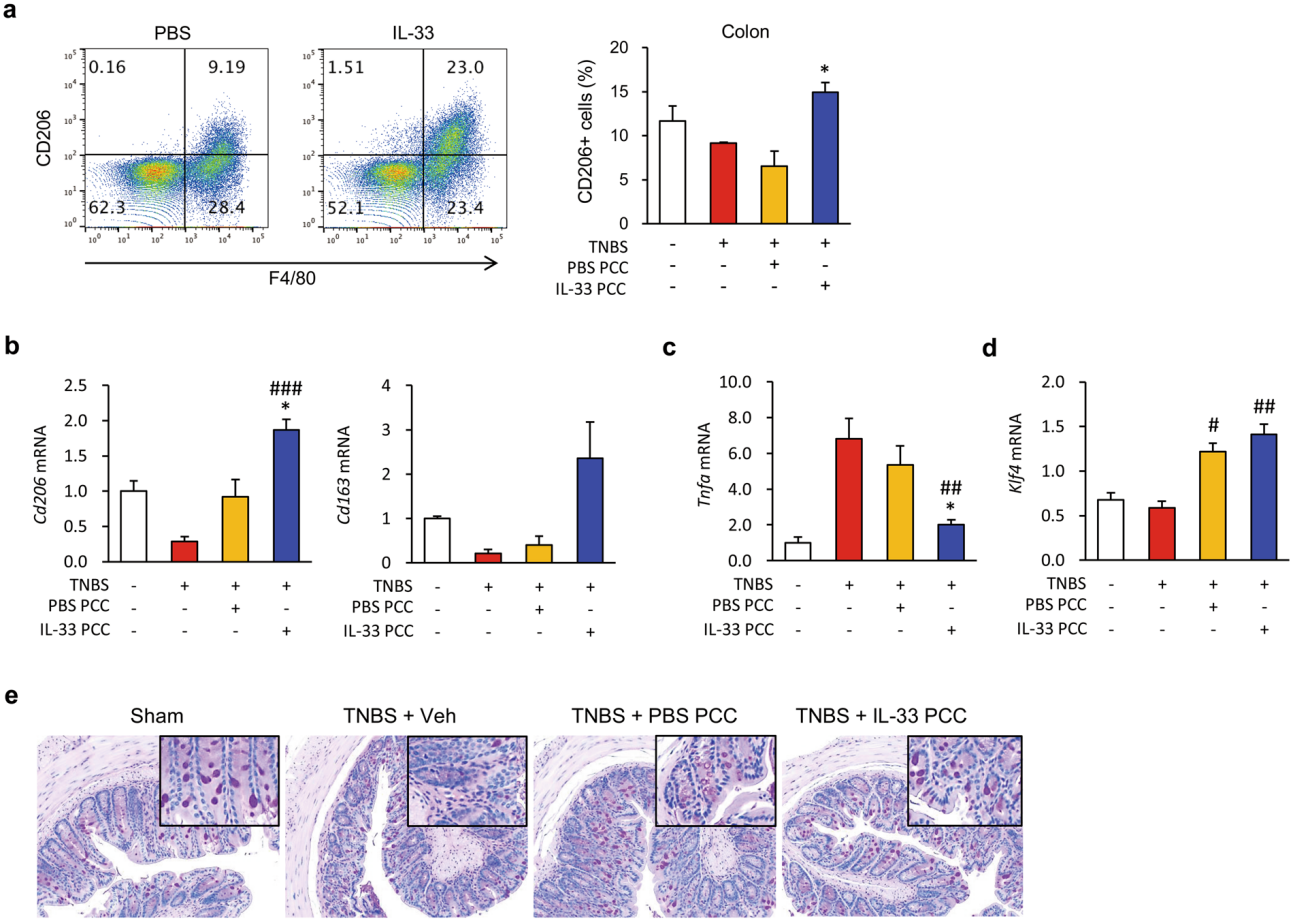
IL-33-treated peritoneal cavity cells rescue M2 macrophages and the goblet cells in colon tissues with colitis. (**a**) Representative flow cytometry plots of M2 (CD206^+^) macrophage (F4/80^+^) populations in colon samples. (**b**–**d**) Effects of IL-33 on the mRNA expression levels of M2 (*Cd206* and *Cd163*, **b**), M1 marker (*Tnfa*, **c**), and goblet cell marker (*Klf4*, D) in the colons. (**e**) Representative periodic acid-Schiff (PAS) staining of colonic mucosal samples. Original magnification: x200. Values are expressed as means (n = 5); error bars represent ±SDs. *P < 0.05 vs. PBS PCC as assessed by ANOVA. PCC, peritoneal cavity cell; Veh, injected with vehicle; IL-33, injected with mrIL-33; TNBS, injected with TNBS.

**Figure 6:**
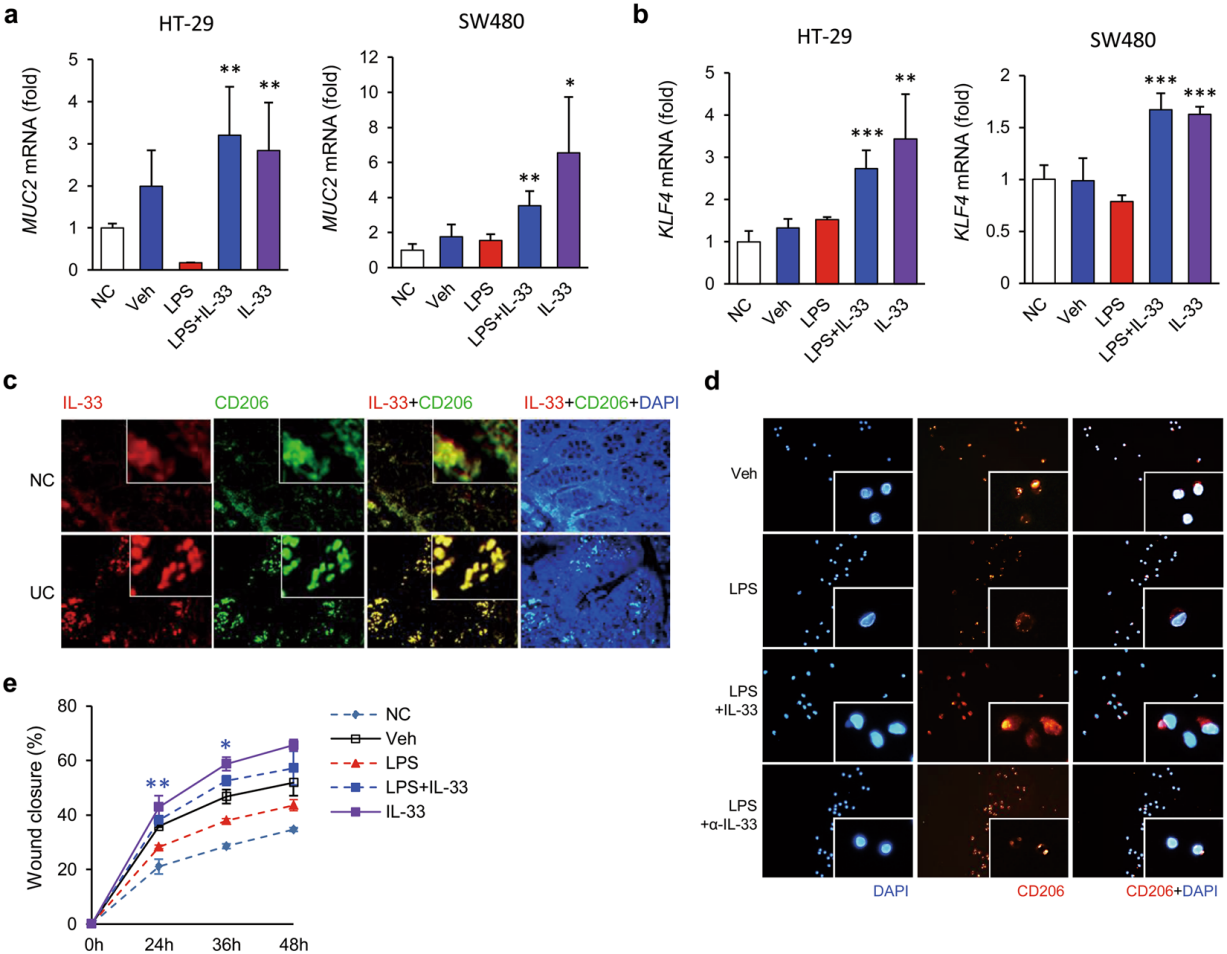
IL-33 drives goblet cell restoration and wound healing via modulation of macrophages toward the M2 phenotype. (**a**,**b**) Effects of IL-33-treated macrophage on goblet cell differentiation. THP-1 monocyte-derived macrophages were treated with vehicle (Veh) or lipopolysaccharide (LPS, 100 ng/ml), with or without IL-33 (20 ng/ml) for 48 hours. Conditioned media from the macrophages were harvested. HT-29 and SW480 cells were treated with normal control media (NC) or the conditioned media for 48 h. Total RNA was extracted from HT-29 and SW480 cells and analysed by quantitative RT-PCR *MUC2* (**a**) and *KLF4* (**b**) mRNA expression levels were normalised to that of *β-actin*. Data are presented as means ± SD (n = 4). (**c**) Co-localisation of IL-33 and CD206 in human colon tissues. Representative images showing that IL-33 is localised to colonic intestinal epithelial cells and lamina propria mononuclear cells. Moreover, IL-33 expression is markedly increased in CD206^+^ cells (M2 macrophages). The immunolocalisation of IL-33 (red) and CD206 (green) in colon tissue samples from normal controls (NC, n = 3) and patients with ulcerative colitis (UC, n = 4) was evaluated by immunohistochemistry using fluorescent conjugated antibodies. Nuclei were counterstained with DAPI (blue). Original magnification: x400. (**d**,**e**) IL-33 drives human monocyte-derived macrophages toward the M2 phenotype and wound repair. Monocytes were isolated from PBMCs (peripheral blood mononuclear cells) and differentiated into macrophages using human recombinant GM-CSF for 7 days. Macrophages were cocultured with the HT-29 cells and wound healing assays were performed. (**d**) Representative images of CD206^+^ cells. Monocyte-derived macrophages from patients with IBD (n = 2) were treated with IL-33 and stained with fluorescent antibodies against CD206 (red). Nuclei were counterstained with DAPI (blue). Original magnification: x400. (**e**) Wound healing assay. Monocytes were isolated from PBMCs and differentiated into macrophages for 7 days. HT-29 cells were cocultured with or without the macrophages (NC) and wound healing assays were performed. Data are presented as means ± SD (n = 3). *P < 0.05 vs. LPS, **P < 0.01 vs. LPS, ***P < 0.005 vs. LPS as assessed by Student’s *t*-test. Veh, treated with vehicle; IL-33, treated with hrIL-33; LPS, treated with lipopolysaccharide; α-IL-33, treated with anti-IL-33 antibodies (IL-33 antagonist).

We next determined if IL-33 directly affects M2 polarisation of macrophages, a process that accelerates wound repair and suppresses proinflammatory cytokines. M2 polarisation has been shown to be potently induced by IL-33 through type 2 cytokines and by regulatory T cells derived from T helper or innate lymphoid cells^36, 37^. Since macrophages express the ST2^38^, we reasoned that IL-33 might directly promote M2 polarisation of macrophages. To test this hypothesis, we peritoneally administered mrIL-33 to naïve mice and isolated PCCs 3 days later. Next, we isolated F4/80^+^ cells from the PCCs using fluorescence activated cell sorting (FACS) and transferred the F4/80^+^ cells to TNBS-treated mice. We found that mrIL-33 administration markedly increased the numbers of CD206^+^ cells isolated from PCCs (Figs 4a and S6a). However, the numbers of Foxp3^+^ cells were only slightly increased and the numbers of Gata3^+^ cells did not exhibit any significant changes compared with the numbers isolated from the spleens of naïve mice (Fig. 4b). Notably, IL-33-F4/80^+^-transferred TNBS-treated mice exhibited significantly better body weight retention, longer colons, and decreased disease severity and histological score (Figs 4c−e and S6b). As expected, IL-33-F4/80^+^ PCC-treated mice exhibited significant increases in the percentages of CD206^+^ cells in the F4/80^+^ populations isolated from the colons compared with PBS-F4/80^+^-treated mice (Figs 5a and S6c). However, unexpectedly, IL-33-F4/80^+^ PCC-treated mice showed modest, but decreases in the numbers of Gata3 (Th2) and Foxp3 (Treg) positive cells in the spleen compared to PBS-F4/80^+^ PCC-treated mice (Fig. 4f). These findings indicate that IL-33 might ameliorate colitis in association with increased M2 macrophages, but irrespective of a Th1-to-Th2 shift or Treg activity. IL-33-F4/80^+^ PCC treatment inhibited the expression of typical M1 markers such as *Tnfa* in the colon tissue samples from TNBS-treated mice, while IL-33-F4/80^+^ PCC treatment induced mRNA expression of typical M2 markers including *Cd206*, *Cd163*, *transforming growth factor beta* (*Tgfb*), and *Il10* (Figs 5b,c and S7a). In particular, IL-33-F4/80^+^ PCC treatment also upregulated *Klf4* and *Muc2* expression (Figs 5d and S7b), suggesting that IL-33 modulates the phenotypes of activated macrophages and recovers the functions of resident enterocytes such as goblet cells. Consistently, histological examinations of the colonic tissue samples revealed significantly reduced colitis and hypotrophic goblet cells in IL-33-F4/80^+^ PCC-treated mice compared with control mice (Fig. 5e). To confirm whether macrophages directly facilitate goblet cell differentiation, we treated mature THP-1 monocyte-derived macrophages with hrIL-33 and LPS, harvested the conditioned media from the macrophage culture after 48 h, and treated HT-29 and SW480 with the conditioned media for 48 h. As shown in Fig. 6a and b, HT-29 and SW480 cells treated with the LPS + hrIL-33-treated conditioned media showed higher *MUC2* expression (4.2 and 2.1 folds, respectively) and, concordantly, higher *KLF4* expression when compared to groups treated with LPS-treated conditioned media (1.5 and 1.4 folds, respectively). Of note, a markedly increased expression of *MUC2* and *KLF4* was observed in the LPS + hrIL-33-treated conditioned media-treated cells than the cells directly treated with LPS + hrIL-33-treated when compared to control groups (1.9 and 2.7 folds in HT-29 cells; 1.8 and 1.5 folds in SW480 cells, respectively). These results indicate that goblet cell modulation by IL-33 might be directly influenced by macrophages, which was first confirmed in this study.

### IL-33 impacts M2 macrophage polarisation from PBMCs of patients with IBD and drives wound healing

To investigate whether IL-33 directly impacts M2 macrophage polarisation in inflamed mucosal samples from patients with IBD, colon tissue sections were coimmunostained with anti-IL-33 and anti-CD206 antibodies. Inflamed colonic tissue sections from patients with UC revealed that mononuclear cells with nuclear staining of IL-33 widely infiltrated into the lamina propria and were not restricted to close proximity to enterocytes. In contrast, healthy colon tissue sections exhibited exclusively close proximity of IL-33 staining (Fig. S8a), consistent with a previous study^4^. Subsequent immunostaining revealed that the IL-33-immunopositive cells coincided with a considerable number of CD206^+^ cells with abundant cytoplasm, characteristics that are typical of tissue macrophages (Figs 6c and S8b). To determine whether IL-33 directly drives macrophages toward the M2 phenotype in humans, we treated monocytes isolated from PBMCs of patients with IBD with granulocyte macrophage-colony stimulating factor (GM-CSF) for 72 h and then with hrIL-33. IL-33 clearly induced CD206^+^ expression in human monocyte-derived macrophages (MDMs), while an IL-33 antagonist completely blocked this induction of CD206^+^ expression (Fig. 6d). Next, we performed a wound healing assay to evaluate the effects of hrIL-33-treated MDMs on wound repair. hrIL-33-treated MDMs were cocultured with scraped (i.e. wounded) HT-29 cells. We found that hrIL-33-treated macrophages mediated significantly greater wound closure in the epithelial cells compared with vehicle-treated macrophages (Figs 6e and S9). In addition, the wound closure rates were higher in control macrophage-treated groups (Veh) than in untreated controls, which is consistent with findings of a previous study that activation of macrophages with LPS induced a concentration-dependent increase the wound healing rate in cultured gastric epithelial cells^39^. These results demonstrate that macrophages play a prominent role in wound healing and that macrophage phenotype plasticity can be modulated by IL-33. Overall, these observations suggest that IL-33 directly induces M2 macrophage polarisation and plays an important role in mucosal wound healing during colitis via the polarisation of M2 macrophages.

## Discussion

Although IL-33 is synthesised as a precursor and can be cleaved by caspase-1 and -3, the cleavage products are biologically less active than their precursor^40^. To rule out the possibility that cleaved forms of IL-33 were undetected in our assays and improve the recently proposed reliability issue of human IL-33 ELISA kit^41^, we used a complementary ELISA kit that analysed the presence of the C-terminal epitope of IL-33, in addition to an ELISA kit used in a previous study^2, 4^. Both kits showed the same results, i.e. serum IL-33 levels were lower in patients with IBD compared with normal control patients. Consistent with the present study, some studies have reported that IL-33 is barely detected in serum samples from patients with UC and CD^3, 4^. However, several other studies have concluded that serum IL-33 levels are elevated in patients with IBD^2–4^. Despite these discrepancies, a general consensus is that IL-33 levels are upregulated in inflamed IBD tissues, especially in UC. As mentioned above, the increased IL-33 levels in the intestine during murine colitis might be due to an endogenous secondary compensatory mechanism to restore the epithelium against tissue damage by increased proinflammatory cytokines such as TNF and IL-1β^2, 42, 43^. Alternatively, they may be due to decreased levels in ST2, a decoy receptor for IL-33, in IECs resulting from active inflammation itself. In this context, recently, genetic studies have shown that IL-33 mRNA levels are not correlated with allele dosage, although genetic polymorphisms in IL-33 contributing to the risk of IBD have been reported^2, 4–6, 44^. The increased sST2 serum levels that have been observed in patients with active UC suggest that sST2 might be involved in controlling the progress of UC, as has been proposed before^4^, although the precise role of sST2 in IBD remains unknown^9^. A particularly interesting finding of our study is that higher serum sST2 levels were associated with more severe disease in patients with UC, although this trend was not significant in patients with CD or intestinal BD. Indeed, high levels of sST2 during chronic intestinal inflammation may further perpetuate pathogenic responses by limiting IL-33-driven Treg accumulation^29^. Moreover, it has also been reported that sST2 mRNA levels are significantly higher in patients with active UC^4^ and that ST2 deficiency results in decreased disease severity in two experimental models of IBD^9^. sST2 can serve as a decoy receptor for IL-33^45^, and conversely IL-33 upregulates sST2 expression, which in turn reduces IL-33 levels^46^, suggesting that sST2 might mask the protective function of IL-33. Additionally, ST2 can blunt macrophage activation by sequestering components in the toll-like receptor 4 (TLR4)/MyD88 pathway, a pathway that is required for IL-33 signalling and gut homeostasis^38, 47^. Taken together, these findings are consistent with our finding that the serum IL-33 levels are reduced in patients with IBD. They also support the idea that reduced IL-33 production in colitis can help amplify and maintain pathogenic responses in the gut. However, we note that some studies have reached conclusions that are not completely in line with ours.

Recently, IL-33 has emerged as an important mediator of mucosal healing and repair in UC and CD^2, 11^. Because of its nuclear localisation sequence, IL-33 has been proposed to act as a chromatin-associated transcriptional regulating factor^23^. Interestingly, nuclear localisation of IL-33 has been observed in IBD^23^, suggesting that IL-33 may directly modulate goblet cell or macrophage differentiation. Consistent with this hypothesis and previous studies^2, 4^, we confirmed that IL-33 is localised in the nucleus in both enterocytes and macrophages of human and mouse colons and that IL-33 induces goblet cell hyperplasia, although further studies are required to test the hypothesis that IL-33 directly regulates genes related to goblet cell differentiation. Since goblet cell depletion is a sign of gut inflammation and a histological criterion for IBD, failure to produce mature goblet cells may compromise mucin production, resulting in a weakened mucosal barrier and chronic gut inflammation elicited by proinflammatory cytokines^48^. Crypt architectural abnormalities accompanied by goblet cell loss have been observed in patients with UC patients with decreased MUC2 production^49^ and IL-33 expression^4^. Consistently, IL-33 has been reported to increase goblet cell hyperplasia^36, 47^; furthermore, IL-33 deficiency depletes goblet cells^42^. However, IL-33 in our study did not exacerbate the disease activity, but ameliorated the colitis. These controversial results suggest that the function of IL-33 in inflammation might be dependent on the balance between IL-33 and the degree and/or type of immune response. Although a previous study concluded that IL-33 induces goblet cell differentiation via a Th2 response and promotes epithelial repair from injury^27^, we did not observe any changes in the splenic population of Th2 cells after IL-33 treatment. A significant proportion of intestinal Foxp3^+^ Treg cells have been shown to coexpress the transcription factor Gata3^28, 29^. Interestingly, IL-23 inhibits the expression of Gata3 and ST2^29^, suggesting that the upregulation of IL-23 in colitis might limit T-cell responsiveness and Treg differentiation in response to IL-33^29^. Such a scenario would partly explain why T-cell responsiveness was reduced in our colitis models. Of note, MyD88-deficient mice showed no difference of goblet cell modulation by IL-33 during colitis when compared with wild-type mice. Taken together, our results indicate that goblet cell modulation by IL-33 might be directly influenced by IL-33 itself or by other non-T cells, such as macrophages, but independently of the MyD88 pathway during colitis. In this context, we found for the first time that the conditioned media from IL-33-treated macrophages enhances more dramatically goblet cell differentiation when compared to IL-33-alone treatment and IL-33-treated F4/80^+^ cell administration modulates goblet cell differentiation *in vivo*. These findings suggest that M2 polarization of macrophages may function as another critical modulator of goblet cells. KLF-4 is a C2H2 zinc-finger containing transcription factor that is highly expressed in the gastrointestinal tract^50^ and required for the terminal differentiation of goblet cells in the colon^35^. Previous studies indicate that suppression of notch signalling increases *Klf4* levels^51^ and IL-33 suppresses notch ligand expression and prevents goblet cell depletion in DSS-induced colitis^12^. Consistently, we demonstrated that IL-33 increases directly and via macrophage *KLF4* expression *in vitro* and *in vivo*, which might lead to restoration of goblet cells by suppression of Notch signalling. Further study to discover a pathway associated with goblet cell modulation by IL-33 during colitis is warranted.

We observed a trend of significantly increased IL-33 expression in colonic mucosal samples and an association of serum sST2 with disease activity in UC but not in CD. These findings might be correlated with the relative predominance of Th2 polarisation and ST2 transcription in UC, in contrast to CD, which is Th1-skewed^4^. In UC, the affected organs accumulate a heterogeneous population of macrophages derived from recruited monocytes. Furthermore, IL-33 can promote macrophage polarisation towards the M2 phenotype in mouse bone marrow-derived macrophages, which enhances type-2 cell-mediated immune responses in airway inflammation^22^. IL-33 has been proposed to promote M2 macrophage development and to enhance TGF-β expression^52^. TGF-β is important because it can also be produced from M2 macrophages and has been proposed to be a major factor responsible for the induction of Tregs^29^. We found that IL-33 induced M2 macrophages in both mouse models of colitis and human MDMs, even though IL-33 did not significantly affect Th2 or Treg populations in the mouse models. Since M2 macrophages derived from M1 macrophages improve the resolution of inflammation and aid wound healing^18^, our results suggest that IL-33 ameliorates colitis via the modulation of macrophages, not of T-cells and, notably, Treg and Th2 cells are dispensable for IL-33-mediated amelioration of colitis in our system, similar to that of a recent studies^14, 53^. Our data suggest that IL-33 ameliorates colitis through its effects on innate immunity rather than adaptive immunity. However, further studies using ST2-deficient mice would be valuable to determine the precise mechanism by which IL-33 induces M2 macrophages.

We focused our present effort on macrophages. In this study, we demonstrated that patients with IBD have lower serum IL-33 levels than healthy controls. Our data suggest that IL-33 facilitates wound healing and ameliorates colitis via modulating the differentiation of goblet cells and M2 macrophages independent of the MyD88 pathway and T-cells. This is the first study to dissect the mechanisms by which IL-33 both directly and indirectly modulates the functions of enterocytes and macrophages in the context of mucosal healing and wound repair during colitis. Our findings, therefore, imply that IL-33 contributes to the resolution of colitis via modulation of macrophages and plays a crucial protective role in IBD.

## Methods

### Study subjects

Forty five healthy subjects, 69 patients with CD, 75 patients with UC, and 74 patients with intestinal BD of Korean descent were recruited at Severance Hospital (Yonsei University College of Medicine, Seoul, Korea) between June 2006 and August 2014. At all times, diagnosis of BD^54^, CD^55^, and UC^55^ was made according to previously established international criteria based on clinical, endoscopic, histopathological, and radiological findings. Patients who were diagnosed or suspected as having indeterminate colitis, coexistence of infectious or ischemic colitis, coexistence of other localised or systemic infections, any malignant diseases, or any major systemic illnesses were excluded. Disease localisation and CD behaviour were described according to the Montreal classification scheme^56^. The CDAI, pMayo, and DAIBD scores were also recorded for each patient at the time of the hospital visit. The control group comprised healthy individuals who had no history of immune-mediated diseases. The demographic and clinical characteristics of the study subjects are summarised in Table S1. This study was approved by the Institutional Review Board of Severance Hospital, Yonsei University (IRB approval number 4-2012-0302). All patients and controls provided written informed consent and all the experiments that involved human subjects were carried out in accordance with the approved guidelines by the IRB.

### Colonic mucosal tissue preparation, monocyte isolation from peripheral blood mononuclear cells (PBMCs), and cell culture

Serum samples were obtained from heparin-treated blood by centrifugation at 1,500 *g* for 15 minutes and stored at −80 °C until processed. Colonic mucosal tissue samples were collected during colonoscopy, immediately snap-frozen in liquid nitrogen, and stored at −80 °C until use. PBMCs were isolated from healthy donors using density gradient centrifugation with Ficoll-Paque Plus (GE Healthcare, Piscataway, NJ, USA). Briefly, cells were spun at 1,000 *g* for 15 minutes. Monocytes were obtained from the remaining cells by magnetic sorting using a human Monocyte Isolation Kit II (Miltenyi Biotec, Bergisch Gladbach, Germany). The obtained monocytes were activated using human recombinant GM-CSF (hrGM-CSF, 100 ng/ml, Promokine, Heidelberg, Germany) and cultured in RPMI1640 containing 10% heat-inactivated foetal bovine serum (FBS, HyClone, Logan, UT, USA) and 1% penicillin/streptomycin (Invitrogen, Carlsbad, CA, USA) for 4 days. Activated monocytes were then divided into the following 7 treatment groups: Veh (vehicle), LPS (1 μg/ml, Sigma-Aldrich, St. Louis, MO, USA), LPS + hrIL-33 (human recombinant IL-33: Ser109-Ile266, 20 ng/ml, Biolegend, San Diego, CA, USA), hrIL-33, α-IL-33 (rabbit anti-human IL-33 antibodies, 1 μg/ml, Santa Cruz Biotechnology, Santa Cruz, CA, USA), and LPS + α-IL-33. After 24 hours, macrophages were detached with a cell scraper (SPL Life Science Inc., Daejeon, South Korea) and analysed by flow cytometry. Alternatively, macrophages were cocultured with HT-29 cells for the *in vitro* wound healing assay or cultured in a confocal dish (SPL Life Science Inc.) for immunofluorescence (IF) staining. HT-29 and SW480, intestinal epithelial cancer cell line (American Type Culture Collection: ATCC), were grown to 80% confluency in RPMI 1640 medium supplemented with 10% FBS and 1% penicillin/streptomycin under standard conditions (humidified atmosphere of 5% CO_2_ at 37 °C). Macrophages were obtained after differentiation of THP-1 cells provided by the American Type Culture Collection (TIB-202TM, ATCC, Rockville, MD, USA). THP-1 cells (ATCC) known to differentiate into macrophages after stimulation with phorbol myristate acetate (PMA, Sigma-Aldrich)^57^ were cultured in RPMI. To obtain macrophages, THP-1 cells were stimulated with PMA (30 nM) for 24 h and washed with PBS 3 times before treatment. Cells were cultured with or without LPS (200 ng/ml; Sigma-Aldrich) for 0, 2, 6, 12, 24, and 48 h. Supernatants were immediately collected, aliquoted, and frozen at −20 °C for later assays; cells were harvested for total protein extraction.

### Determination of serum concentrations of IL-33 and soluble receptor ST2 (sST2)

Serum IL-33 and sST2 concentrations were determined in duplicate using ELISA kits to detect the C-terminal epitope of IL-33 (LEGEND MAX™ Human IL-33 ELISA Kit, BioLegend; DuoSet IL-33 ELISA kit, R&D Systems, Minneapolis, MN, USA) and sST2 (R&D Systems), respectively, in accordance with the manufacturer’s recommended protocols. Briefly, serum samples were incubated in 96-well plates that had previously been coated with anti-IL-33 and sST2-specific monoclonal antibodies for 2 hours at room temperature. After washing the wells four times, peroxidase-conjugated polyclonal antibodies against IL-33 and sST2 were added. Samples were incubated for 2 hours at room temperature followed by four washes, after which the substrate solution was added. After incubation for 30 minutes at room temperature, the reactions were terminated by the addition of stop solution. The optical density of each well at 450 nm was determined using a VersaMax ELISA Microplate Reader (Molecular Devices, Sunnyvale, CA, USA). All values were corrected by normalising to the corresponding absorbances at 540 nm. All data were analysed with SoftMax Pro 6.3 (Molecular Devices).

### DSS, TNBS, and PCC transfer animal studies

To induce acute colitis, 8–9-week-old male C57BL/6 mice were administered 3% (w/v) DSS (MP Biomedicals, Solon, OH, USA) in their drinking water. The first day of administration was designated day 0; DSS was administered for 6 consecutive days. The mice received i.p. mouse recombinant IL-33 (mrIL-33, 0.2 μg/mouse, BioLegend) or PBS daily from days 1 to 5. On day 7, the day before sacrifice, the drinking water with DSS was replaced with plain drinking water. For TNBS-induced colitis, mice were lightly anaesthetised with 2 mg/kg Zoletil (Virbac Laboratories, Carros, France) and 10 mg/kg xylazine (Rompun, Hver-Lockhart Laboratories, Shawnee Mission, KS, USA) by i.p. injection. A total of 100 µl TNBS solution (1.8% (w/v) for mild colitis, 2.2% (w/v) in the PCC and survival models; Sigma-Aldrich) in 40% ethanol (Sigma-Aldrich) was injected into the colon of the mouse via the anus using a 1 ml syringe equipped with a round thin-tip needle. After insertion into the colon approximately 4 cm from the anus, the mice were hung in a head-down position for 2 min to allow the administered agents to be distributed entirely throughout the colon and caecum. The normal group was treated with vehicle only. The mice were evaluated daily for body weight loss, the presence of gross blood in the faeces or at the anus, stool consistency and overall mortality. A validated DAI score previously used in our study^58^ was calculated for each mouse before sacrifice. The score ranged from 0 to 4 and was calculated based on the following parameters: weight loss (0, none; 1, 1–5%; 2, 5–10%; 3, 10–20%; 4, >20%), gross bleeding (0, absence; 2 and 3, light bleeding; 4, bleeding), and stool consistency (0, negative; 1 and 2, loose; 3 and 4, diarrheal). DAI scores were expressed as the average of these three parameters. The severity of colitis was assessed by an independent observer who was blinded to mouse assignments. Mouse recombinant IL-33 (mrIL-33, 1 μg/mouse) or sterilised PBS (vehicle) was injected into naive mice for the PCC transfer model and for the other TNBS model. In the PCC transfer model, PCCs were extracted from the peritoneum of each mouse by washing the peritoneal cavity with cold PBS at 3 days post-IL-33 i.p. injection. From these harvested cells, F4/80 positive cells were sorted using a FACSAria flow cytometer (BD Bioscience, San Jose, CA, USA). Next, 1 × 10^6^ sorted cells were i.p. injected into the recipient mice. After 2 days, the recipient mice were lightly anaesthetised and injected with TNBS solution. At 4 days post-TNBS treatment, which corresponded to 7 days post-DSS administration, the mice were euthanised and the entire colon (from the cecum to the anus) was quickly removed from each mouse. The colon length from the ileo–caecal junction to the proximal rectum was measured, after which a longitudinal incision was made along the entire colon and all stool was eliminated by washing with PBS. Subsequently, the distal colon was divided into 3 portions for PAS staining, immunofluorescence, and RNA isolation. All recipient mice were fed a standard diet until reaching the age at which the experiment began. All animal experiments were performed according to all applicable Korean laws and were reviewed and approved by the Institutional Animal Care and Use Committee of Yonsei University Severance Hospital, Seoul, Korea (IACUC Approval No: 2013-0166) and were carried out in accordance with the approved guidelines by the IACUC.

### Isolation of macrophages from the colon and isolation of lymphocytes from the spleen

Lamina propria mononuclear cells (LPMCs) were isolated from the harvested mouse colons as previously described^59^. Briefly, each spleen was cut into small fragments. The fragments were passed through a 70-mm pore cell strainer (BD Bioscience) into a 60 mm culture dish. The dish contained 4 ml RPMI 1640 medium supplemented with 10% heat-inactivated foetal bovine serum and 1% antibiotics. Next, the tissue fragments were crushed using the plunger of a 2 ml syringe and the crushed tissues were harvested. After depletion of red blood cells with RBC lysis buffer, the remaining cells were analysed by flow cytometry.

### PAS staining, histology, and immunofluorescence

Animal and human tissue samples were fixed with 10% formalin solution (pH 7.4) overnight, embedded on a slide with paraffin and sectioned using standard protocols. Sections were then deparaffinised, incubated in warm 1% citrate buffer for 10 min, cooled at room temperature for 1 hour and then washed with distilled water for 2 min before antigen retrieval. Nonspecific binding sites were blocked by incubation in 1% BSA/PBS for surface molecule staining; to detect intracellular components, blocking was performed with 1% BSA/PBS with 0.1% Triton X-100. Subsequently, rabbit anti-human IL-33 (1:200, Santa Cruz) and FITC-conjugated CD206 (1:50, BD Bioscience) antibodies were added to the sections and incubated at 4 °C overnight. IL-33 signal was detected with goat anti-rabbit IgG Alexa Fluor 555 antibodies (1:500, Invitrogen). Nuclei were counterstained with DAPI (blue). Images were obtained using a fluorescence light microscope (Olympus BX41, Olympus Optical, Tokyo, Japan).

### Flow cytometry

Cell suspensions were prepared in PBS containing 2% FBS. Cells (1 × 10^6^) were blocked with mouse Fc blocking solution (anti-mouse CD16/CD32 mAb 2.4G2, BD Bioscience) and stained for 30 minutes at 4 °C with the appropriate antibodies. The antibodies used included: human and mouse anti-Foxp3 (1:50, 150D/E4) and anti-Gata3 (1:50, TWAJ) (eBioscience; San Diego, CA, USA); mouse anti-CD3 (1:50, 145-2C11), anti-CD4 (1:50, GK1.5) (BD Bioscience; F4/80), anti-BM8 (eBioscience), and anti-CD206 (1:50, C068C2) (BioLegend); and human anti-CD3 (1:50, SK7), anti-CD206 (1:50, 19.2) (BD Bioscience), anti-CD4 (1:50, OKT4), and anti-CD11b (1:50, ICRF4) (eBioscience). For intracellular staining, a Foxp3/Transcription factor staining buffer set was purchased from eBioscience. Data were acquired using a FACSVerse flow cytometer (BD Bioscience) and analysed using FlowJo software (Tree Star, San Carlos, CA, USA).

### Extraction of RNA and quantitative real-time RT-PCR

Total RNA was extracted using TRIzol Reagent (Invitrogen) and reverse transcribed using a SuperScript First-Strand Synthesis Kit (Invitrogen) according to the manufacturer’s protocol. Next, 20 ng of RNA was reverse-transcribed using a SuperScript First-Strand Synthesis kit (Life Technologies) according to the manufacturer’s protocol. Reactions contained cDNA, SYBR Green Master Mix (Applied Biosystems, Foster City, CA, USA) and the appropriate primer pair; all reactions were set up in triplicate. Amplification was performed using a StepOne Plus real-time PCR system (Applied Biosystems) for 45 cycles using the following thermocycling steps: 95 °C for 30 seconds, 60–63 °C for 30 seconds, and 72 °C for 40 seconds. Quantitative PCR (qPCR) primers are summarised in Table S2. Finally, quantitative analysis was performed using the relative comparative method using the following equation: relative gene expression = 2^−(ΔCt sample−ΔCt control)^, and the results were reported as the fold change as compared to the calibrator or 2^ΔCt^ after normalization of the transcript level to the endogenous control.

### *In vitro* wound healing assay

HT-29 colon epithelial cells were cultured in 12-well plates under standard culture conditions until reaching 80% confluence. Next, wounds were created using a plastic tip and images were captured using a fluorescence microscope. The plates were marked to facilitate the identification of the same region at later time points. Next, monocytes cultured together with lymphocytes were added to the wounded HT-29 cells (5 × 10^5^ cells/well). To estimate the degree of wound healing, images were captured every 12 hours and the percentage closure was calculated using cellSens software (Olympus Optical).

### Statistical analysis

Associations between clinical and laboratory parameters and serum IL-33/ST2 levels were analysed by calculating Pearson’s correlation coefficients. All results are expressed as means of 3–5 samples ± standard deviation (SD) or ± standard error of mean (SEM). Prism software (GraphPad Software, Inc., San Diego, CA) was used for all analysis. The significance of differences between conditions was assessed using Student’s *t*-test and one–way ANOVA. *P* values < 0.05 were considered significant. *P < 0.05, **P < 0.005, and ***P < 0.001 compared to the control condition.

## Electronic supplementary material

Supporting Information
