# Supplementary material for: Interleukin-33 regulates intestinal inflammation by modulating macrophages in inflammatory bowel disease
Source: Sci Rep. 2017 Apr 12;7:851. doi: 10.1038/s41598-017-00840-2 (PMC5429815; doi:10.1038/s41598-017-00840-2)
Supplement: Supplementary file 1 — Supporting Information [file 41598_2017_840_MOESM1_ESM.pdf]

Supporting Information

Interleukin-33 Regulates Intestinal Inflammation by Modulating Macrophages in Inflammatory Bowel Disease

Dong Hyuk Seo, BS,[1,2,3*] Xiumei Che, MS,[1,2,3*] Min Seob Kwak, MD,[4,5*] Soochan Kim, PhD,[1,3] Jae Hyeon Kim, BS,[1,2] Hyun Woo Ma, BS,[1,2] Da Hye Kim, MS,[1,3] Tae Il Kim, MD, PhD,[1] Won Ho Kim, MD, PhD,[1] Seung Won Kim, PhD,[1,2,3†] and Jae Hee Cheon, MD, PhD,[1,2,3†]

[1]Department of Internal Medicine and Institute of Gastroenterology, Yonsei University College of Medicine, Seoul, Korea

[2]Brain Korea 21 PLUS Project for Medical Science, Yonsei University College of Medicine, Seoul, Korea

[3]Severance Biomedical Science Institute, Yonsei University College of Medicine, Seoul, Korea

[4]Department of Internal Medicine, Graduate School, Yonsei University College of Medicine, Seoul, Korea

[5]Department of Internal Medicine, Kyung Hee University Hospital at Gang Dong, Kyung Hee University School of Medicine, Seoul, Korea

[*,†]These authors contributed equally to this work

Supplementary Tables

Table S1. Patient clinical characteristics

Characteristic	CD (69)	UC (75)	BD (74)	NC (45)
Age, yr, median (IQR)	23.5 (8.6)	34.0 (21.0)	41.0 (18.0)	35.0 (2.0)
Sex, (%)				
Male	51 (73.9)	44 (58.7)	29 (39.2)	19 (47.5)
Female	17 (24.6)	31 (41.3)	45 (60.8)	21 (52.5)
UC extent, (%)				
Proctitis		13 (17.3)		
Left sided colitis		45 (60.0)		
Extensive colitis		17 (22.7)		
Location, (%)				
L1 (ileal)	14 (20.3)			
L2 (colonic)	15 (21.7)			
L3 (ileocolonic)	26 (37.7)			
L4 (only upper GI)	4 (5.8)			
NA	10 (14.5)			
Behaviour at diagnosis, (%)				
B1 (nonstricturing, nonpenetrating)	16 (23.2)			
B2 (stricturing)	25 (36.2)			
B3 (penetrating)	18 (26.1)			
NA	10 (14.5)			
CDAI, median (IQR)	94.3 (51.9)			
pMayo score, median (IQR)		2.0 (3.0)		
DAIBD, median (IQR)			20.0 (31.0)	
Serum IL-33 level, median (ng/ml, IQR)	0.09 (0.38)	0.07 (0.10)	0.07 (0.12)	0.16 (0.37)
Serum sST2 level, median (ng/ml, IQR)	5.11 (1.56)	5.76 (1.52)	5.38 (1.47)	4.50 (0.99)
ESR, mm/h, median (IQR)	30.0 (44.5)	23.0 (33.0)	27.0 (22.0)	
CRP, mg/dL, median (IQR)	1.8 (7.0)	2.3 (6.0)	1.4 (7.0)	

IQR, interquartile range; GI, gastrointestinal; NA, not applicable; pMayo, partial Mayo scoring index; CDAI, Crohn's disease activity index; DAIBD, disease activity index for intestinal Behcet`s disease; NC, normal control.

Table S2. **Primers for qRT-PCR**

	Forward	Reverse
Mouse		
Arg1	CTCCAAGCCAAAGTCCTTAGAG	AGGAGCTGTCATTAGGGACATC
β-Actin	AGTGTGACGTTGACATCCGT	TGCTAGGAGCCAGAGCAGTA
Cd163	GGTGGACACAGAATGGTTCTT	CCAGGAGCGTTAGTGACAGC
Cd206	CAGGTGTGGGCTCAGGTAGT	TGTGGTGAGCTGAAAGGTGA
Il10	GCCACATGCTCCTAGAGCTG	CAGCTGGTCCTTTGTTTGAAA
Il33	TCCAACTCCAAGATTTCCCCG	CATGCAGTAGACATGGCAGAA
Klf4	AGAGGAGCCCAAGCCAAAGAGG	CCACAGCCGTCCCAGTCACAGT
Muc2	GGTCCAGGGTCTGGATCACA	GCTCAGCTCACTGCCATCTG
Tgfb1	ACCATGCCAACTTCTGTC	CGGGTTGTGTTGGTTGTAGA
Tnfa	CAAAGGGAGAGTGGTCAGGT	ATTGCACCTCAGGGAAGAGT
Human		
BACTIN	CTCTTCCAGCCTTCCTTCCTG	CAGCACTGTGTTGGCGTACAG
IL33	CACCCCTCAAATGAATCAGG	GGAGCTCCACAGAGTGTTCC
KLF4	CGGACATCAACGACGTGAG	GACGCCTTCAGCACGAACT
MUC2	AGGATGACACCATCTACCTCACC	GGTGTAGGCATCGCTCTTCTC

Supplementary Figures

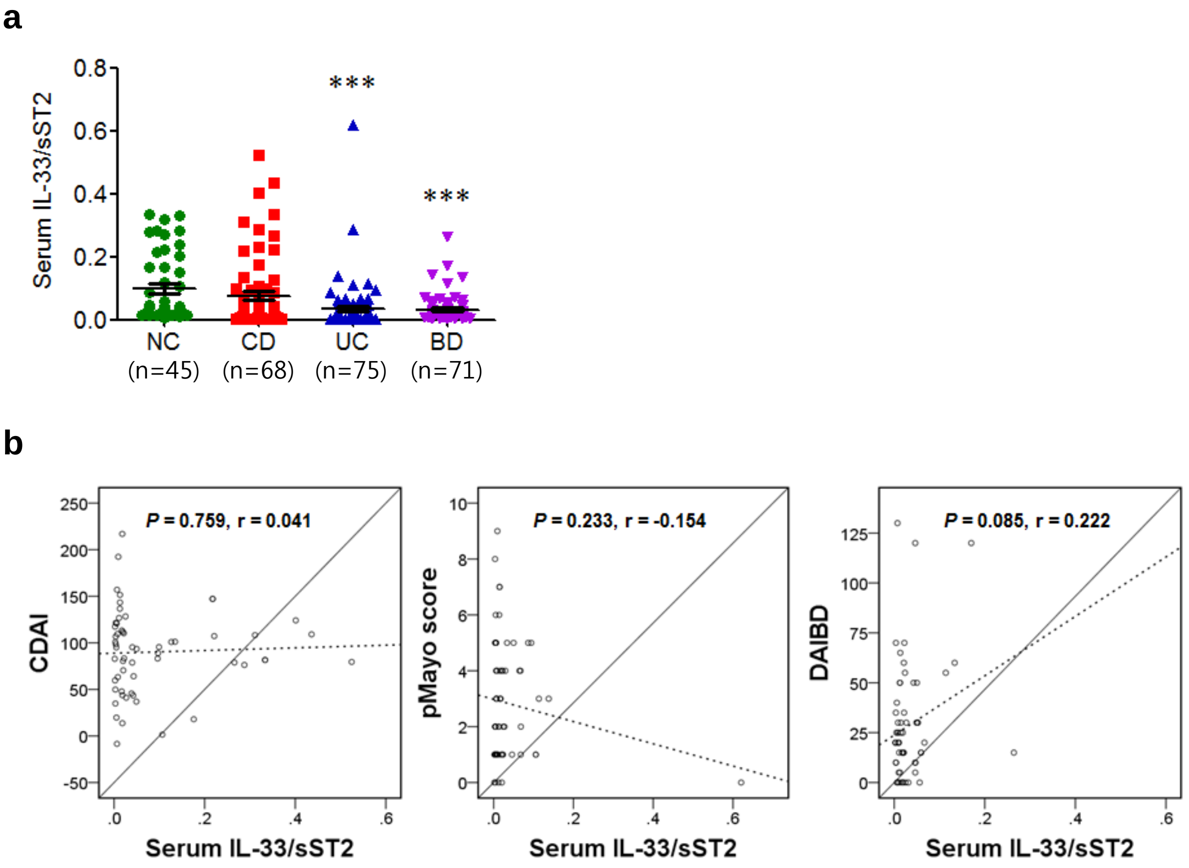

Figure S1. Ratios of circulating IL-33/ST2 and its correlation with disease activity. (**a**) Serum IL-33/sST2 ratios in patients with inflammatory bowel disease (IBD). (**b**) Correlation between serum IL-33/sST2 ratio and IBD clinical disease activity. Solid line, references on the X and Y axes; dotted line, fit for total variables. Results are shown as individual values (symbols) and SDs (lines) for each group of patients. ****P* < 0.005 vs. NC as assessed by ANOVA. NC, normal control; CD, Crohn's disease; CDAI, CD activity index; UC, ulcerative colitis; BD, Behçet’s disease; DAIBD, disease activity index for intestinal BD.

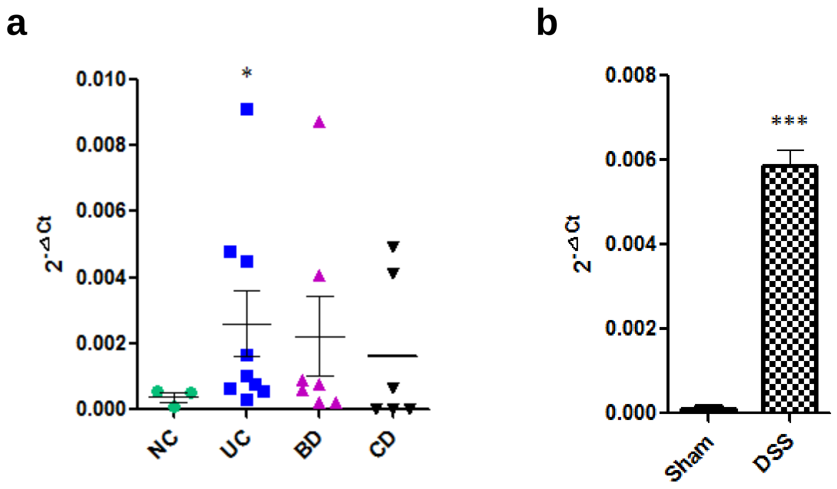

Figure S2. IL-33 mRNA expression in inflamed mucosal tissue samples. (**a**) Colonic biopsy samples were harvested from noninflamed areas of normal control (NC, n = 3) patients and from inflamed areas of patients with UC (n = 9), BD (n = 7), and CD (n = 6). Total RNA was extracted and processed for quantitative RT-PCR. IL-33 expression levels were normalised to that of β-actin. Relative mRNA levels were calculated using the $2^{\Delta Ct}$ formula. The significance of differences between groups was analysed using Student's *t*-test. (**b**) Effects of IL-33 on dextran sodium sulfate (DSS)-induced colitis in mice (n = 5 mice/group). $^{*}P < 0.05$ vs. NC, $^{*}P < 0.05$ vs. Sham. NC, normal control; UC, ulcerative colitis; BD, Behçet's disease; CD, Crohn's disease; Veh, injected with vehicle; IL-33, injected with mrIL-33; TNBS, injected with 2,4,6-trinitrobenzenesulfonic acid.

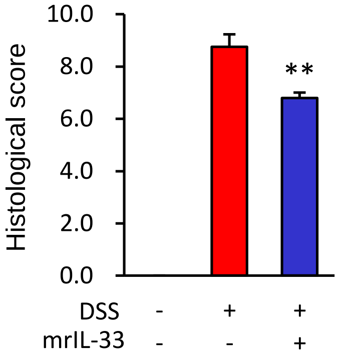

Figure S3. Histological score of dextran sodium sulfate (DSS)-induced colitis in mice (n = 5). $^{**}P < 0.01$ vs. DSS. IL-33, 5 daily injections of IL-33 (mrIL-33, 0.2 μg/mouse); DSS, treated with 3% (w/v) DSS.

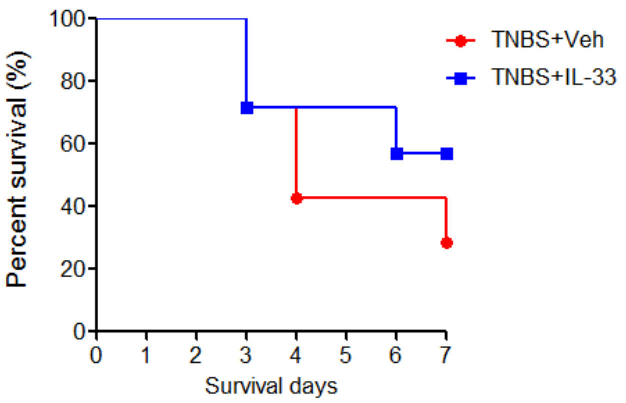

Figure S4. Effects of IL-33 on the survival of mice with 2,4,6-trinitrobenzenesulfonic acid (TNBS)-induced colitis. Survival (n = 7) was analysed by generating a Kaplan-Meier plot. Veh, treated with vehicle; IL-33, treated with mrIL-33; TNBS, injected with 2,4,6-trinitrobenzenesulfonic acid.

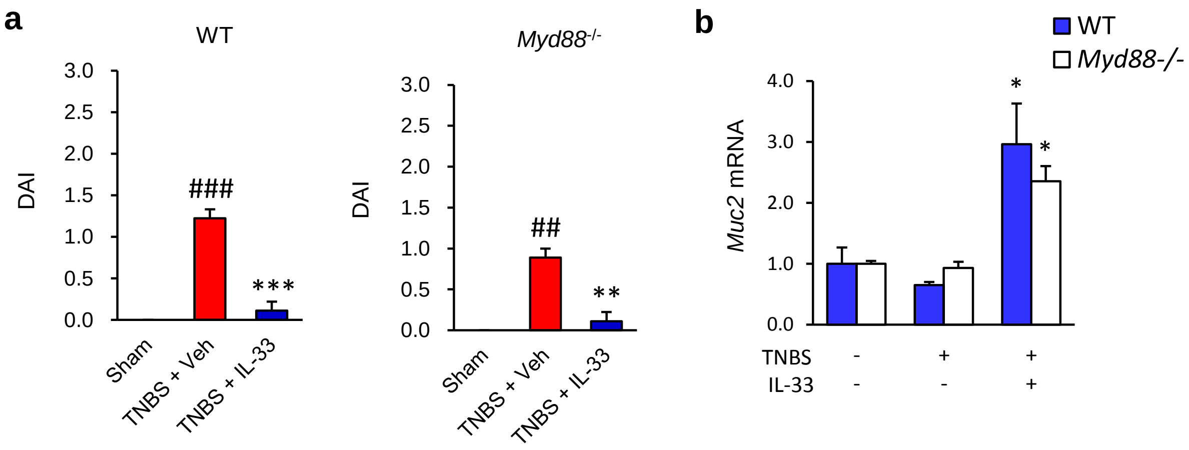

Figure S5. Effects of IL-33 on disease activity index (DAI, a) and Muc2 expression in colon tissue (b) of wild-type (WT) and Myd88 deficient (*Myd88*$^{-/-}$) mice with 2,4,6-trinitrobenzenesulfonic acid (TNBS)-induced colitis (n = 5). To assess the effects of IL-33 on enterocyte and macrophage early in disease, 100 µl TNBS solution (1.8% (w/v)) in 40% ethanol was injected into the colon of each mouse and DAI score at day 6 (D6) was obtained. $^{\#\#}P < 0.01$ vs. Sham, $^{\#\#\#}P < 0.005$ vs. Sham, $^{**}P < 0.01$ vs. TNBS, $^{***}P < 0.005$ vs. TNBS. Veh, treated with vehicle; IL-33, treated with mrIL-33; TNBS, injected with 2,4,6-trinitrobenzenesulfonic acid.

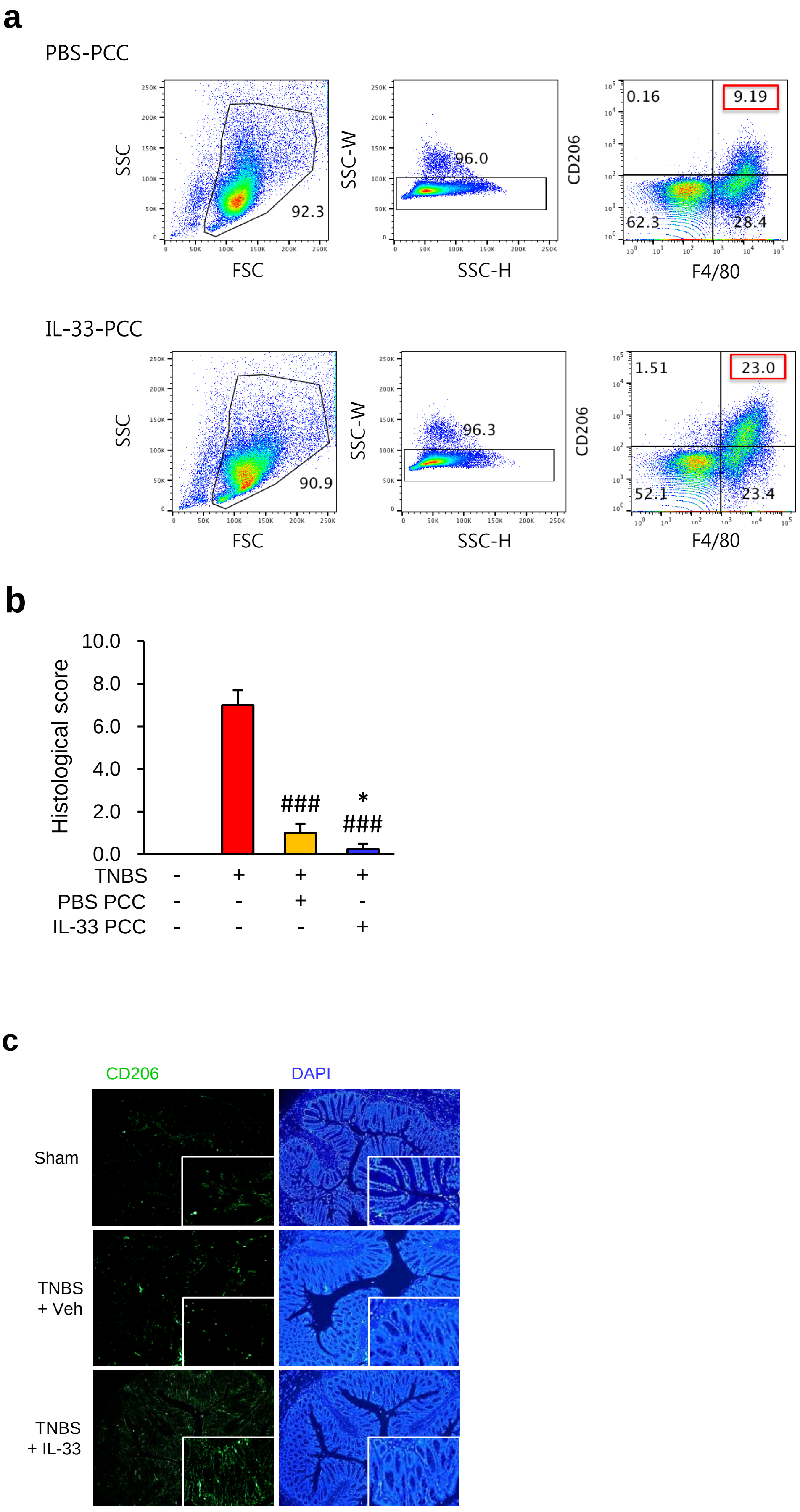
a
PBS-PCC
SSC
FSC
92.3
SSC-W
SSC-H
96.0
CD206
F4/80
0.16
9.19
62.3
28.4
IL-33-PCC
SSC
FSC
90.9
SSC-W
SSC-H
96.3
CD206
F4/80
1.51
23.0
52.1
23.4
b
Histological score
10.0
8.0
6.0
4.0
2.0
0.0
###
*
###
TNBS - + + +
PBS PCC - - + -
IL-33 PCC - - - +
c
CD206
DAPI
Sham
TNBS + Veh
TNBS + IL-33

Figure S6. Mouse PCC transfer model. Male C57BL/6 mice were inoculated i.p. with IL-33 (1 μg/mouse) or PBS for 3 consecutive days (**a**). Next, peritoneal cavity cells (PCCs) were extracted from the peritoneum of each mouse by washing the peritoneal cavity with cold PBS. F4/80 positive cells were sorted from the extracted PCCs and i.p. injected (1×10^6 cells) into the recipient mice. After 2 days, 2,4,6-trinitrobenzenesulfonic acid (TNBS) solution was injected into the colon of each recipient mouse (**b**,**c**). (**a**) Representative flow cytometry gating strategies of peritoneal cavity cells (PCCs) isolated from PCC-treated mice (n = 5). (**b**) Histological score of the colon from TNBS-treated mice. Values are expressed as means (n = 5); error bars represent ±SDs. (**c**) Representative image of mouse colon immunostaining (n = 3–4). CD206 is visualized in green using fluorescent conjugated antibodies. Nuclei were counterstained with DAPI (blue). $^{###}P < 0.05$ vs. TNBS, $*P < 0.05$ vs. PBS PCC as assessed by ANOVA. Veh, treated with vehicle; IL-33, treated with mrIL-33; TNBS, injected with 2,4,6-trinitrobenzenesulfonic acid.

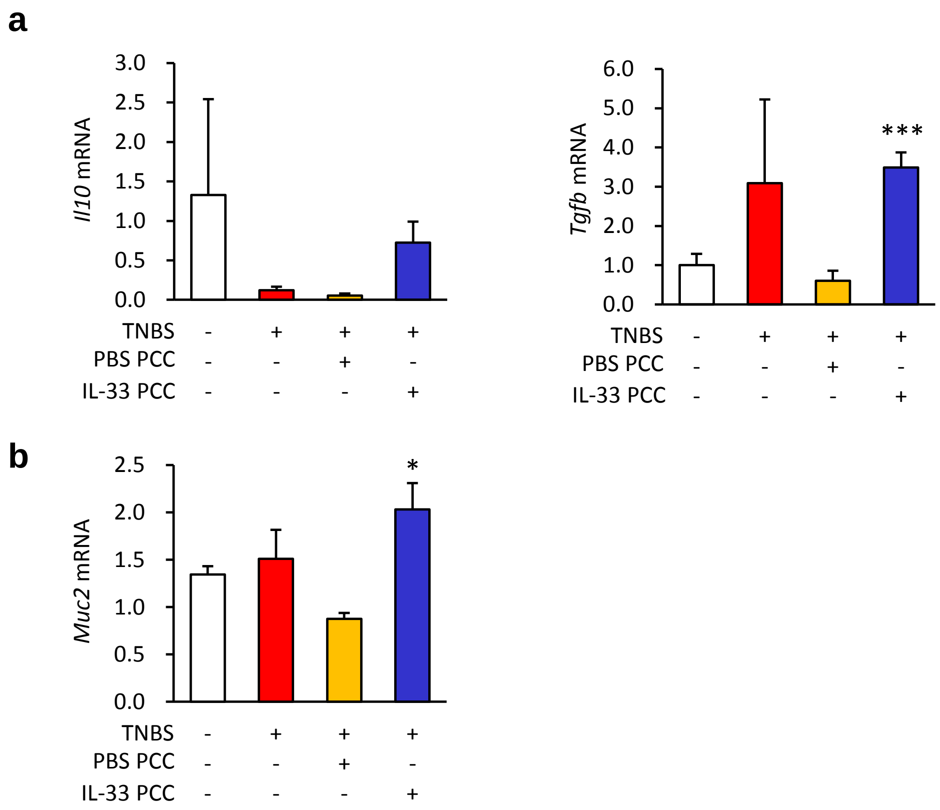

Figure S7. Effects of IL-33 on the mRNA expression levels of M2 and goblet cell markers in the colons from peritoneal cavity cell (PCC)-treated mice. (**a**) mRNA expression of *Il10* and *Tgfb*. (**b**) mRNA expression of *Muc2*. Male C57BL/6 mice (n = 5) were inoculated i.p. with IL-33 (1 μg/mouse) or PBS for 3 consecutive days. Next, peritoneal cavity cells (PCCs) were extracted from the peritoneum of each mouse by washing the peritoneal cavity with cold PBS. F4/80 positive cells were sorted from the isolated PCCs and i.p. injected (1×10^6 cells) into the recipient mice. After 2 days, 2,4,6-trinitrobenzenesulfonic acid (TNBS) solution was injected into the colon of each recipient mouse. ****P* < 0.005 vs. PBS PCCs as assessed by ANOVA. Veh, treated with vehicle; IL-33, treated with mrIL-33; TNBS, injected with 2,4,6-trinitrobenzenesulfonic acid.

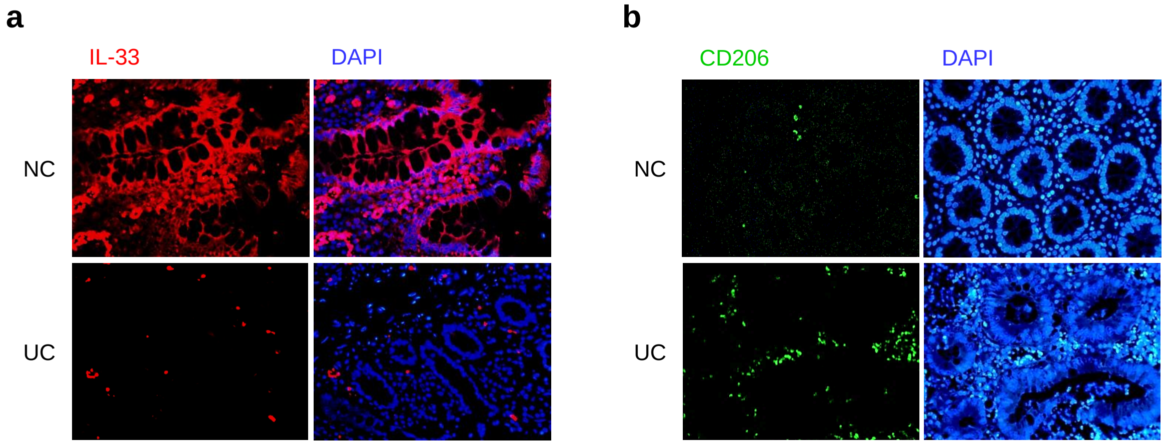

Figure S8. Representative image of CD206 and IL-33 immunostain in human colon (NC, n = 3; UC, n = 4). (**a**) CD206 immunostaining (green). (**b**) IL-33 immunostaining (red). Nuclei were counterstained with DAPI (blue). NC, normal control; UC, ulcerative colitis.

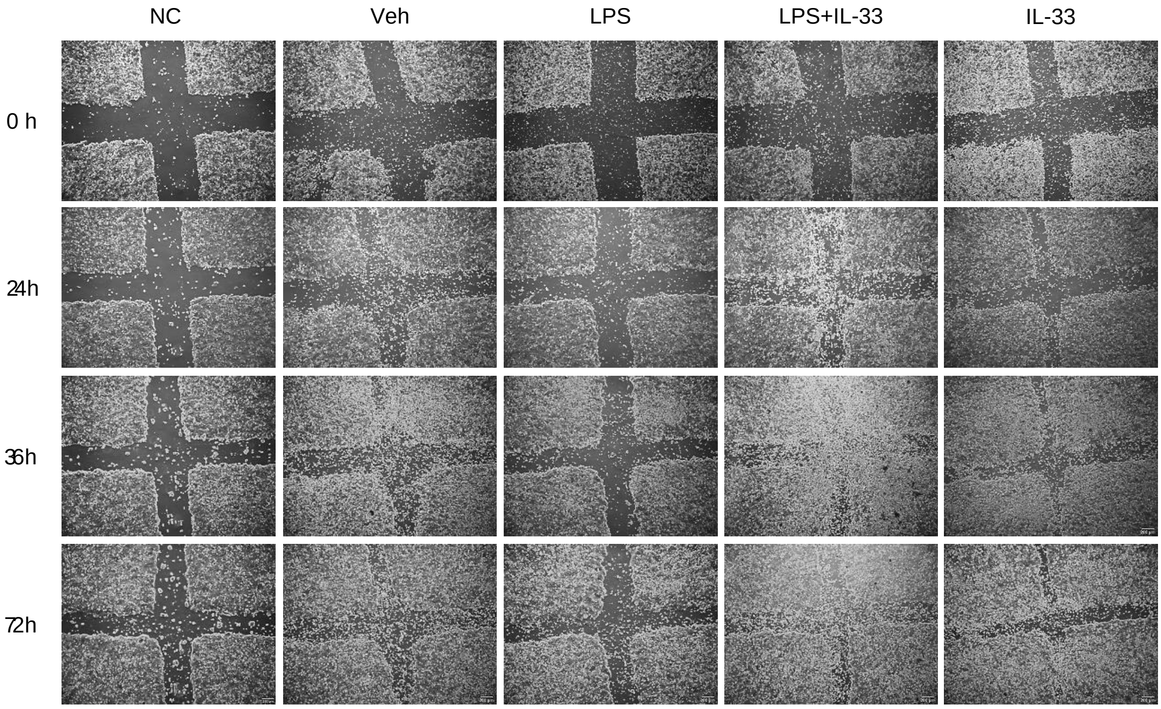

Figure S9. Wound healing assay. Monocytes were isolated from PBMCs and differentiated into macrophages for 7 days. Macrophages were cocultured with the HT-29 cells and wound healing assays were performed. Representative images show increased wound closures by IL-33 treatment. Data are presented as means ±SD (n = 2). *P < 0.05 vs. Veh as assessed by Student's *t*-test. NC, not cocultured; Veh, treated with vehicle; IL-33, treated with hrIL-33; LPS, treated with lipopolysaccharide. $^{*}P$ < 0.05 vs. LPS.
